# Research on eye health 2000–2019: a global bibliometric analysis with a focus on equity

**DOI:** 10.1136/bmjophth-2025-002404

**Published:** 2026-02-02

**Authors:** Jacqueline Ramke, Saad Chugtai, Justas Bezaras, Lisa M Hamm, David Macleod, Jinfeng Zhao, Iris Gordon, Jennifer R Evans, Matthew Burton

**Affiliations:** 1 International Centre for Eye Health, London School of Hygiene and Tropical Medicine, London, UK; 2 School of Optometry and Vision Science, The University of Auckland, Auckland, New Zealand; 3 ApaJee Trust, Nowshera, Khyber Pakhtunkhwa, Pakistan; 4 Kaunas University of Technology, Kaunas, Lithuania; 5 Department of Infectious Disease Epidemiology and International Health, London School of Hygiene and Tropical Medicine, London, UK; 6 Department of Epidemiology and Biostatistics, University of Auckland, Auckland, New Zealand; 7 National Institute for Health Research Biomedical Research Centre for Ophthalmology at Moorfields Eye Hospital NHS Foundation Trust and UCL Institute of Ophthalmology, London, UK

**Keywords:** Public health

## Abstract

**Objective:**

To summarise global peer-reviewed primary research on eye health published from 2000 to 2019.

**Methods and analysis:**

We used the ‘explode eye disease’ function on MEDLINE to obtain all articles reporting primary research studies on eye health published between 1 January 2000 and 31 December 2019. We were intentionally broad and included population, clinical, animal and laboratory studies. We categorised the main eye condition of the paper from Medical Subject Headings (MeSH) terms, and the country of the study from the first country listed in the abstract (or if this was absent, the affiliation of the first author). A validated algorithm was used to assign gender to authors.

**Results:**

We included 158 697 publications from 178 countries. Across the period, annual research output increased globally (4.2% per annum, 5057 publications in 2000 to 10 875 in 2019) and in 20 of 21 regions. There was substantial geographical maldistribution, with research output ranging from 1.0 publication/million population in Central Sub-Saharan Africa to 165.8/million in Australasia; 70% of research identified was conducted in high-income countries (n=1 11 417). 42% of publications focused on one of the five leading causes of vision impairment. Of the 789 463 authorships assigned a gender, women held 33% of all (n=261 636/789 463), 36% of first (n=47 729/131 664) and 24% of last authorships (n=31 720/129 800). Women formed 50% of authorship teams when the last author was a woman (IQR 38–71%), compared with 20% of teams when the last author was a man (IQR 0–40%).

**Conclusion:**

The annual research output doubled globally over the two decades, with a disproportionate output from high-income countries and slow progress towards gender parity. The main limitations of our study included the use of a single database, which may have led to an underestimation of all outputs, particularly from low- or middle-income countries.

**WHAT IS ALREADY KNOWN ON THIS TOPIC**

- Previous bibliometric analyses on eye health research have tended to focus on specific geographical regions, conditions or journals.

**WHAT THIS STUDY ADDS**

- During the two decades of *Vision 2020: Right to Sight*, there was an increase in the number of publications reporting primary eye health research. Over two-fifths of all research focused on one of the five leading causes of vision impairment; disparity between outputs from high-income and middle-income or low-income countries and between male and female authors continued, though some improvements occurred.

**HOW THIS STUDY MIGHT AFFECT RESEARCH, PRACTICE OR POLICY**

- This study provides a summary of the primary research studies in eye health undertaken globally during the period of the *Vision 2020: Right to Sight* global initiative, which was also the period just prior to the release of WHO’s inaugural *World Report on Vision* and the *Lancet Global Health* Commission on Global Eye Health.
- Ongoing monitoring of research output will help inform the global response to the recommendation in the *World Report on Vision* for more evidence to inform integrated people-centred eye care.

## Introduction

 In its inaugural *World Report on Vision* in 2019, the World Health Organization (WHO) recognised the value of evidence for eye health, and called for high-quality research to inform the implementation of integrated people-centred eye care.^1^ The analysis presented here formed part of the *Lancet Global Health* Commission on Global Eye Health.^2^ The Commission reviewed the *Vision 2020: The Right to Sight* initiative, which concluded in 2020 following two decades of significant achievement in global eye health. This milestone, alongside the launch of the *World Report on Vision*, warranted multifaceted reflections, including on the full extent of primary research conducted over the Vision 2020 period. Previous bibliometric analyses in eye health have tended to focus on shorter periods,^3^ or specific geographical regions,^4^ conditions^5 6^ or journals.^7^,^10^

Eye health is not experienced equally by everyone. In 2020 it was estimated there were 596 million people living with blindness or moderate or severe vision impairment, 90% of whom lived in low- or middle-income countries.^2 11^ The regions with the highest age-standardised prevalence of blindness were Western and Eastern sub-Saharan Africa, while the regions with the highest number of people living with vision impairment or blindness were South Asia and East Asia.^2^ Within countries, women, people living in rural areas and Indigenous peoples are often among the population groups underserved by existing services.^2^ Three-quarters of vision loss in 2020 was due to five causes: uncorrected refractive error, cataract, glaucoma, macular degeneration or diabetic retinopathy.^12^

Unfortunately, there is a dearth of evidence on how to deliver more effective and equitable eye health services^13^ and a much greater emphasis on equity by eye health researchers is required.^2^ A recent analysis of 1.5 million medical studies showed that research teams with a higher proportion of women were more likely to undertake gender and sex analysis,^14^ providing evidence that more diverse research teams would likely promote equity in eye health research.^15^

Our aim for the analysis presented here was to undertake a bibliometric assessment of peer-reviewed articles reporting primary studies on eye health published between 2000 and 2019 with respect to eye condition, where research was done and the inclusion of women in authorship.

## Materials and methods

### Search and study selection

Our search was constructed by an experienced information specialist (IG) on MEDLINE in July 2020 and updated in October 2021 to allow for any delay in Medical Subject Headings (MeSH) coding of 2019 studies. The search was intentionally broad and had no language restrictions to capture as much of the eye literature as possible (box 1). Drawing on the broad definition of eye health outlined by the Lancet Commission as ‘maximised vision, ocular health and functional ability’,^2 16^ we aimed to include any article reporting primary research on an eye-related topic, which could include animal research and basic laboratory research. All fields for each included record were exported from MEDLINE into Microsoft Excel for analysis.

Box 1 **Summary of search to construct sample**

1. exp eye diseases/
2. limit 1 to yr=“2000–2019”
3. case reports/
4. 2 not 3
5. limit 4 to journal article
6. limit 5 to (meta analysis or “review” or “systematic review” or systematic reviews as topic)
7. 5 not 6
8. limit 7 to (address or autobiography or bibliography or biography or comment or editorial or “expression of concern” or festschrift or interview or lecture or letter or news or patient education handout or periodical index or personal narrative or portrait or technical report or webcast)
9. 7 not 8

### Data preparation and analysis

*Data cleaning and preparation:* We undertook some additional cleaning steps to arrive at our final data set. We undertook deterministic deduplication using DOI (digital object identifier) when available, and otherwise title and first author (n=689). Due to our interest in articles reporting primary research, we searched for the following terms in the title or abstract and excluded such records that made it through the initial search (box 1): bibliography, case report, editorial, erratum, guideline, literature review, practice guideline, retraction, republished, reprint (n=1435). We also removed records with neither MeSH terms nor an abstract as these tended to be letters or journal business (n=5180). To prevent parsing errors, we removed invalid special characters from the data set (eg, stray symbols like ‘ï»¿’).

*Main eye condition:* The list of MeSH headings and sub-headings under ‘eye disease’ was used to categorise each record to one of the following conditions: (1) cataract, (2) refractive error, (3) glaucoma, (4) age-related macular degeneration (AMD), (5) diabetic retinopathy, (6) corneal condition, (7) trachoma or (8) other condition. The following criteria were used:

1. If a paper only had MeSH terms for one of the conditions, it was coded to that condition.
2. If a paper had MeSH terms from more than one of the conditions, it was assigned to the condition that had the greater number of terms. For example, if a paper had three diabetic retinopathy terms and one glaucoma term, it was assigned to diabetic retinopathy.
3. We had planned to assign any paper with an equal number of MeSH terms from two or more conditions to the condition that caused the higher magnitude of global blindness in 2020 (ordered from one to seven above) but this was not required.
4. If a paper had no MeSH terms related to conditions 1 to 7, it was coded as ‘other condition’.

The proportion of research in each super-region undertaken on each condition was calculated.

*Location of study:* The location of the research was determined using three approaches. The country of affiliation of the first author was extracted and separately, the name of any country in the abstract was extracted. Where only one of these was available, it was used to assign the location of the research. Where the country of affiliation differed from the country in the abstract, the country in the abstract was used. Where more than one country was listed in the abstract, the first country listed was used. When a country was not indicated in the affiliation or abstract, the place of publication was used (∼3% of records). Each country name was standardised (eg, converted ‘UK’ to ‘United Kingdom’) and assigned to the relevant Global Burden of Disease (GBD) region and super-region (online supplemental table 1).^17^ The regional per capita research output was calculated using the total number of articles identified between 2000 and 2019 divided by the regional population in 2015; this was plotted against the age-standardised prevalence of blindness in each region.^11^ Mean annual percentage increase was estimated using a Poisson regression, with number of publications as the outcome and year as the exposure. These estimates were solely used as descriptive indicators of change over time, not for statistical inference, and therefore CIs were not reported.

*Gender of authors:* MEDLINE began to record full names of all authors from 2002, so the analysis on gender excluded articles published in 2000 and 2001. The position of all authors (first, middle, last) and their first name and surname was extracted. We used a validated algorithm (gender-api.com) to assign gender (male/female/unknown) to authors based on their first name. For each article, the gender of the first author and last author was recorded, and the proportion of all authors who were women was calculated. Within each region and globally, we calculated the median, IQR and range of the proportion of authorships per article held by women, disaggregated by the gender of the senior author. Across all articles, we calculated the average proportion of all authors, first authors and senior authors who were women for each region in each year. Change over time in the proportion of female authors (all, first and last) was estimated using a generalised linear model with a binomial distribution and identity link, using gender as the outcome and publication year as the exposure. As before, these were used descriptively, and statistical inference was not performed. To assess the potential impact of unclassified authorship gender on our findings, we conducted a sensitivity analysis modelling three scenarios, whereby all unknowns were (1) assigned male, (2) split evenly between male and female and (3) assigned female.

## Results

We identified 166 001 publications reporting primary research on eye health topics between 2000 and 2019 and after applying our additional cleaning (n=7304 removed) we ultimately included 158 697 publications from 178 countries for analysis.

### Location of research

**Figure 1:**
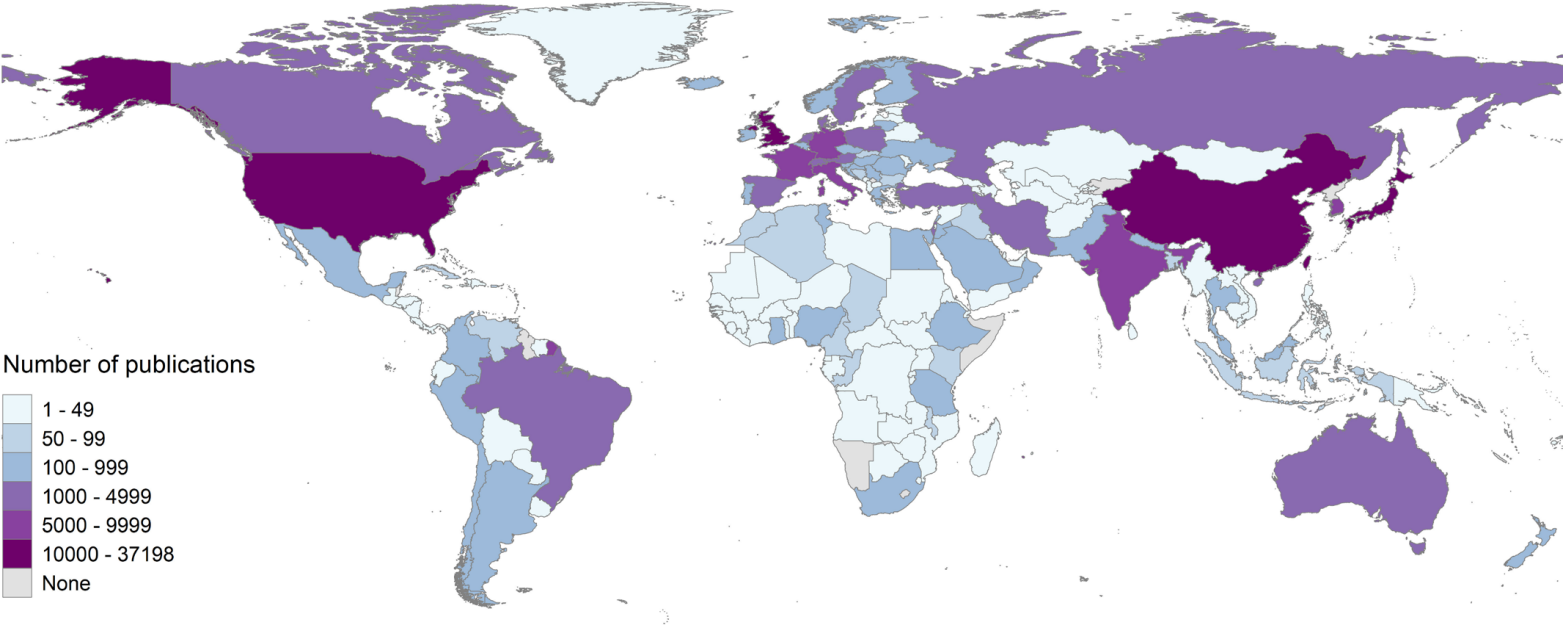
Global distribution of articles reporting primary research studies on eye health published between 2000 and 2019. Studies identified by ‘explode eye disease’ on MEDLINE, July 2020 and again in October 2021; n=158 697.

The countries with the highest output across the time period were the USA (n=37 198, 23% of all publications), China (n=14 414, 9%), Japan (n=10 827, 7%) and the UK (n=10 248, 6%) (figure 1). There was substantial maldistribution in the geographical focus of eye health research, with 70% of published reports originating from high-income countries (n=111 417). At the global level, using the total output across the 20-year period, there was 21.6 publications on eye health/million population. The regional output varied from 165.8 publications/million population in Australasia down to 1.0 /million population in Central sub-Saharan Africa. The per capita research output across regions tended to reduce as the age-standardised prevalence of blindness increased (online supplemental figure 1; online supplemental table 2).

### Change over time

**Figure 2:**
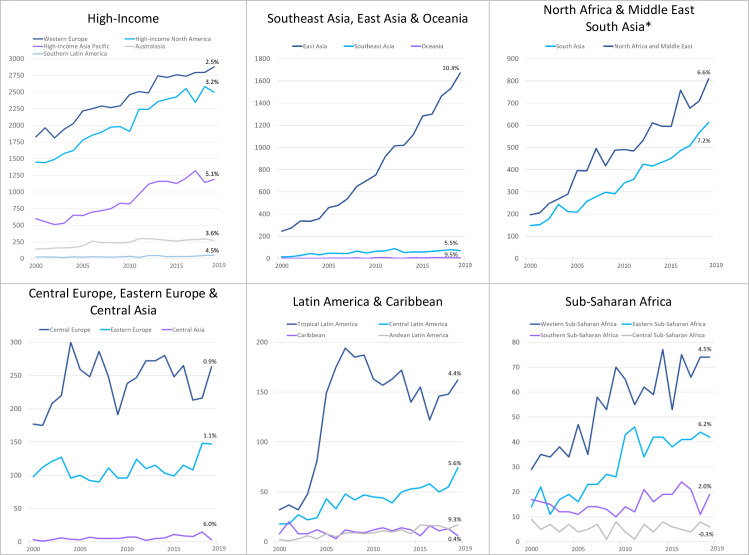
Eye health research output in each GBD region 2000–2019, organised by annual research output. *South Asia and North Africa and Middle East are regions and super-regions—shown together here. GBD, Global Burden of Disease.

At the global level, the annual research output doubled between 2000 and 2019, from 5057 publications to 10 875 (figure 2, online supplemental table 3), with an annual increase of 4.2%. The increase was greatest in absolute terms in high-income countries (from 4041 in 2000 to 6888 in 2019) and South-east Asia, East Asia and Oceania (from 263 in 2000 to 1750 in 2019, primarily driven by increases in China). The regions with the highest mean percentage increase in annual output across the time period were East Asia (10.3%), Oceania (9.5%), Andean Latin America (9.3%) and South Asia (7.2%). Of the 21 regions, the three with the smallest annual percentage increase were Central Europe (0.9%), Caribbean (0.4%) and Central sub-Saharan Africa (0.3% decrease).

### Condition

**Figure 3:**
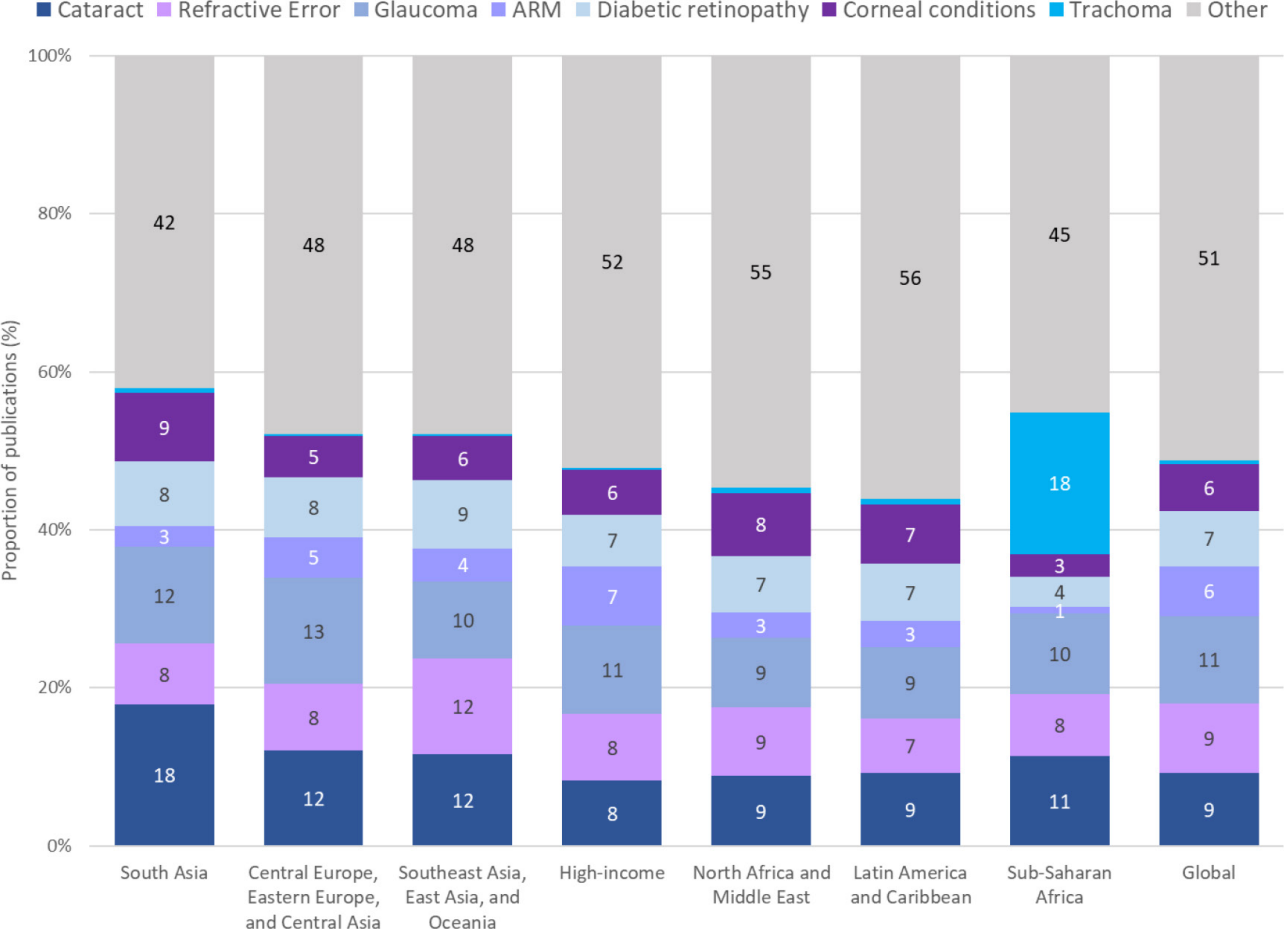
Main condition of primary research on eye health by GBD region, 2000–2019. ARM, age-related macular degeneration; GBD, Global Burden of Disease.

Almost half of all included publications (n=67 196, 42%) were on one of the five leading causes of vision impairment. Glaucoma was the condition most frequently researched at the global level (n=17 409, 11%), followed by cataract (n=14 716, 9%) and refractive error (n=13 958, 9%) (online supplemental table 4, online supplemental figure 2). The region with the largest focus on these leading causes was South Asia (n=3336/6863, 49%) while sub-Saharan Africa was the region with the least focus (n=723/2124, 34%) (figure 3, online supplemental table 5). Indeed, in sub-Saharan Africa, trachoma was more often the focus of a publication compared with any of the five leading causes of global vision impairment (n=381/2124, 18%). South Asia was the region with the highest proportion of publications focused on cataract (n=1227/6863, 18%) while Southeast Asia, East Asia and Oceania was the region with the highest proportion of research on refractive error (n=2141/17 593, 12%).

### Authorship

Across the 146 677 articles from 2002 to 2019, there were 888 658 authorships and the algorithm could assign gender to 789 463 of these (89%); >90% of the 99 195 unassigned people was due to the record having initials instead of a name. Overall, women held 33% of all authorships assigned a gender (n=261 636/789 463), 36% of first authorships (n=47 729/131 664) and 24% of last authorships (n=31 720/129 800). There was regional variation, with women tending to be more included in authorship teams in research undertaken in the regions of Central Europe, Eastern Europe and Central Asia (42%, 11 058/26 431) and Latin America and Caribbean (39%, 7878/20 132), and least included in South Asia (29%, 9048/30 934) and sub-Saharan Africa (28%, 2666/9477) (online supplemental table 6a–d).

**Figure 4:**
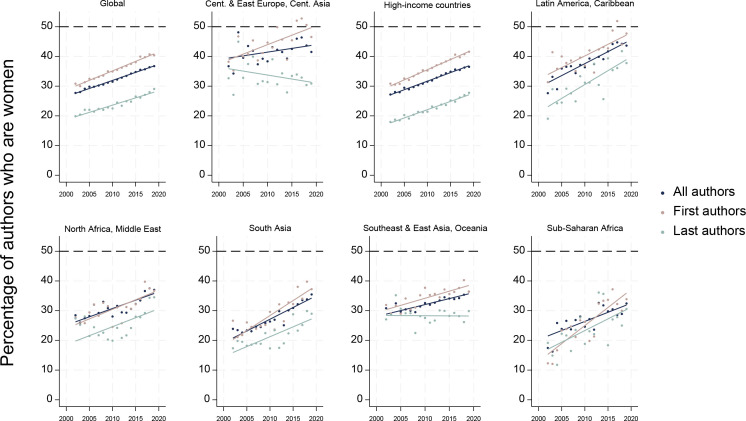
Proportion of all authors, first authors and last authors of eye health research 2002–2019 that were women.

Between 2002 and 2019, the proportion of authorships across all articles that were held by women increased from 28% (5935/21 415) to 37% (23 344/63 531) globally; women as first authors increased from 31% (1351/4381) to 40% (3801/9415) and as last authors increased from 20% (862/4338) to 29% (2769/9528) (figure 4, online supplemental figure 6a–d). If the average annual global increase from this period continued at the same rate (0.54%, 0.64% and 0.49% per annum for all, first and last authors, respectively), it will take approximately 25, 15 and 43 years, respectively, for gender parity to be realised.

For all authorships and first authorships, the gradual increase in women over time was fairly consistent across regions, with the highest average annual increase in proportion of all authors who were women seen in Latin America and Caribbean (0.80% per annum) and South Asia (0.79% per annum) and for first authors in sub-Saharan Africa (1.21% per annum). Gender parity was only observed among first authorship in Central Europe, Eastern Europe and Central Asia (in several years) and in Latin America and the Caribbean since 2016 (figure 4). The pattern for female last authorship was less consistent, though the proportion of women last authors was higher in 2019 than 2002 for all regions except Central Europe, Eastern Europe and Central Asia (33% in 2002 (82/251) and 31% in 2019 (105/340)). Average annual improvement in women as last authors was greatest in Latin America and Caribbean (0.93% per annum). By 2019, women were last authors of 28% high-income country articles (1693/6094), which was the worst performing region.

**Figure 5:**
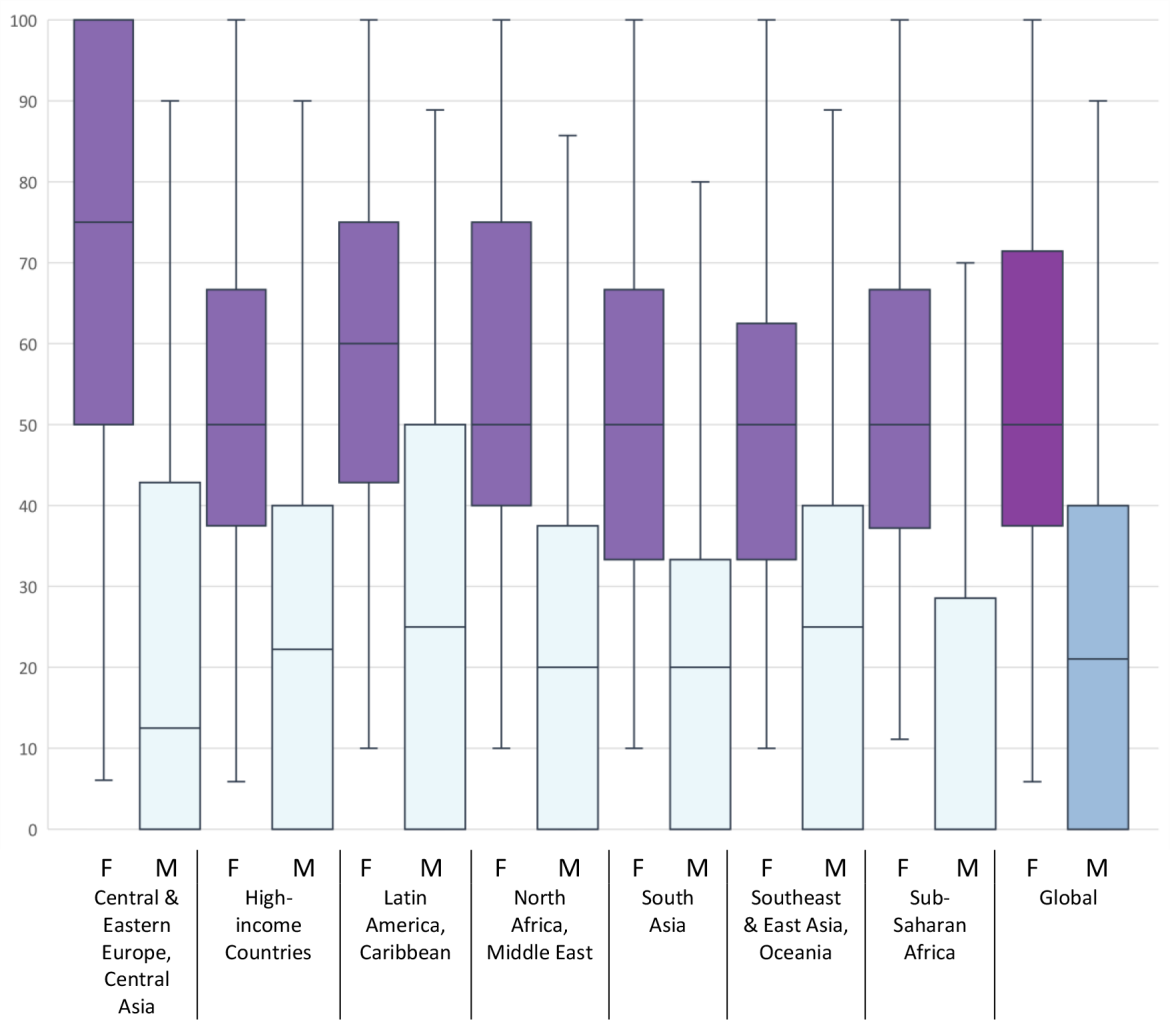
Proportion of research teams who are women based on the gender of last author across Global Burden of Disease super-regions, primary eye health research 2002–2019 (n=128 167 publications). Boxplots show median, first and third quartiles, minimum and maximum. F, last author female; M, last author male.

There was a clear gender disparity based on the gender of the senior author, with women holding 50% of authorships when the last author was a woman (IQR 38–71%), compared with 20% of authorships when the last author was a man (IQR 0–40%) (figure 5, online supplemental table 7). These findings were fairly consistent across regions, though women were more likely to be involved in authorship teams of a female last author in Central Europe, Eastern Europe and Central Europe (75%, IQR 50–100%) and Latin America and Caribbean (60%, IQR 43–75%). When men were the senior author, at least a quarter of articles involved no women coauthors globally, and in all regions. The gender disparity based on the senior author was greatest in sub-Saharan Africa, where at least half the teams with a male senior author contained no women (median female authorships 0%; IQR 0–29%).

In sensitivity analysis regarding the 99 195 authorships (11%) with unassigned gender, the proportion of authorships held by women would reduce from 33% to 29% if all unassigned authorships were male, and would increase to 35% or 41% if half or all the unassigned authorships were female, respectively (online supplemental table 8).

## Discussion

We have taken a broad approach to summarise the research undertaken globally in eye health in the 20 years to 2020. We found an increase in output over time, particularly in East Asia. More than two-thirds of research was undertaken in high-income countries, home to only 14% of the global population and 10% of people with vision impairment.^11^ Furthermore, there was more than a 100-fold difference in research output/capita between high-income regions with the highest output and the regions of Southeast Asia and Central sub-Saharan Africa with the lowest per capita output.

Across the two decades, we identified the annual increase of research outputs globally was 4.2%, with highest output from Western Europe and North America, which aligns with previous studies^3 18^; much of the increase in outputs was driven by increased output from China. Our use of the 21 GBD regions allowed a more nuanced assessment of regional differences and reinforces the call for increased research capacity strengthening in regions currently under-represented in terms of research output, particularly those settings with a high magnitude of vision impairment.^2^ Fortunately, several under-represented regions showed substantially greater relative increase in output over the two decades, though there are still massive disparities compared with the absolute number of outputs compared with Western Europe and North America. Continued emphasis must be placed on the structural barriers faced by research in low-resource settings, including limited research funding and constrained opportunities for international collaboration.^2^

It is encouraging that just over two-fifths of research (42%) was undertaken on one of the five leading causes of vision impairment. However, we were unable to ascertain the extent to which this research addressed a relevant question for eye health services in settings where most vision impairment occurs. For example, cataract and refractive error together cause 90% of distance and near vision impairment^12^ and were considered in 18% of research outputs we identified in this study. However, if the majority of refractive error research is on laser surgery, this will have limited impact addressing the challenge of hundreds of millions of people being unable to access good quality refractive error services. Furthermore, there are several examples in eye health that suggest much more investment is made in quantifying problems than developing and evaluating solutions.^19^,^21^ Ideally, in future, there would be an increasing emphasis on solution-focused research questions and funding^22^ to maximise the proportion of research that answers questions relevant to policymakers and patients.^1 2^

Our results on female authorship in eye health research and increases over time align with previous findings in the ophthalmology literature,^23^,^30^ while providing a broader and more global picture. By not restricting our search to specific article types, journals or English language, we provide a global picture of the field, which highlights progress has been better in some regions than others. Given our analysis predates the COVID-19 pandemic and the corresponding disparities experienced by women,^31^ an updated analysis may reveal that progress towards gender parity in eye health publishing has slowed or reversed.^32^ Regardless, at current rates of progress, we remain decades away from gender parity.

The gender disparity in research teams assembled by men was consistent across all world regions, and particularly concerning was the finding that at least one in every four authorship teams led by men contained no women. The ability of women to assemble teams in which at least 50% of members are female reinforces the fallacy of the commonly used ‘pipeline problem’ to explain lower inclusion of women in science and medicine^33^—for parity to be achieved, the reasons why men have not tended to include women in their research teams must be addressed, alongside the promotion of women to more senior positions. Hopefully, the increased attention and many systemic solutions identified recently can accelerate progress towards parity.^33^,^35^

Our findings must be interpreted in the context of several limitations. For practical reasons, we used only one database (MEDLINE) to identify the records included in this analysis. While using MEDLINE to identify records that have been MeSH coded as an eye disease or condition is a robust approach,^3^ we cannot rule out potential misclassification of conditions during indexing^36^ or by our classification approach, and we may have under-estimated the overall eye health research output for the period by not including additional databases such as Embase which includes journals not indexed in MEDLINE,^37^ and Wanfang Data and other databases focused on languages other than English. Conversely, we may have over-estimated the overall research output in two main ways. First, while we excluded various publication types at the search strategy stage and undertook additional cleaning steps, we cannot rule out the possibility that some of our included articles were not reports of primary research. Second, by using the MeSH term ‘exp eye disease’ we retrieved relevant references across a broad range of topics and ophthalmic conditions, but we likely also retrieved records where the eye component is an adverse event or side effect rather than the main focus of the research. For the gender analysis, there were ∼100 000 names (11%) that could not be assigned a gender, almost exclusively due to only an initial being indexed rather than a full name. In addition, the algorithm has a slight tendency to over-allocate names as male, but these two limitations are unlikely to change our overall interpretation of the result.^38^ Finally, while not a limitation of our approach per se, we recognise that there are many other disparities in research teams beyond country and gender/sex that cannot be analysed with historical data. We are encouraged by recent efforts to standardise data collection in publishing to monitor other social axes along which disparity exists, including race/ethnicity.^39^

## Conclusion

During the two decades when the global *Vision 2020: Right to Sight* initiative was implemented, there was an increase in the number of publications reporting primary eye health research, with over two-fifths of all research focused on one of the five leading causes of vision impairment. Disparity between outputs from high-income and middle-income or low-income countries and between male and female authors continued, though some improvements occurred. To reduce persistent disparities, future efforts should prioritise strengthening research capacity in under-represented regions and promoting solution-focused studies that address the most pressing eye health needs. Aligning research agenda with policy priorities and service delivery challenges will be critical to achieving equitable and effective eye health globally. Ongoing monitoring of research output may help inform the global response to the recommendation in the *World Report on Vision* for more evidence to inform integrated people-centred eye care.

## Supplementary material

10.1136/bmjophth-2025-002404 online supplemental file 1

## Data Availability

Data underlying the figures are included in the supplemental material. Other data are available upon reasonable request.
