# Supplementary material for: Research on eye health 2000–2019: a global bibliometric analysis with a focus on equity
Source: BMJ Open Ophthalmol. 2026 Feb 2;11(1):e002404. doi: 10.1136/bmjophth-2025-002404 (PMC12878186; doi:10.1136/bmjophth-2025-002404)
Supplement: online supplemental file 1 [file bmjophth-11-1-s001.pdf]

Supplementary Table 1: Countries listed by GBD Super Regions and GBD Regions

We have categorised countries according to the Global Burden of Disease (GBD) Super Regions (7) and GBD Regions (21) as outlined by the Institute for Health Metrics (2017). These are listed here:

Super-region	Region and country
**Central Europe, Eastern Europe and Central Asia**	**Central Europe:** Albania, Bosnia and Herzegovina, Bulgaria, Croatia, Czech Republic, Hungary, Macedonia, Montenegro, Poland, Romania, Serbia, Slovakia, Slovenia<br>**Eastern Europe:** Belarus, Estonia, Latvia, Lithuania, Moldova, Russian Federation, Ukraine<br>**Central Asia:** Armenia, Azerbaijan, Georgia, Kazakhstan, Kyrgyztan, Mongolia, Tajikistan, Turkmenistan, Uzbekistan
**High-income**	**Australasia:** Australia, New Zealand<br>**High-income Asia Pacific:** Brunei Darussalam, Japan, South Korea, Singapore<br>**High-income North America:** Canada, United States<br>**Southern Latin America:** Argentina, Chile, Uruguay<br>**Western Europe**: Andorra, Austria, Belgium, Cyprus, Denmark, Finland, France, Germany, Greece, Iceland, Ireland, Israel, Italy, Luxembourg, Malta, Netherlands, Norway, Portugal, Spain, Sweden, Switzerland, United Kingdom
**Latin America and Caribbean**	**Andean Latin America:** Bolivia, Ecuador, Peru<br>**Caribbean:** Antigua and Barbuda, Bahamas, Barbados, Belize, Cuba, Dominica, Dominican Republic, Grenada, Guyana, Haiti, Jamaica, Puerto Rico, Saint Lucia, Saint Vincent and the Grenadines, Suriname, Trinidad and Tobago<br>**Central Latin America:** Colombia, Costa Rica, El Salvador, Guatemala, Honduras, Mexico, Nicaragua, Panama, Venezuela<br>**Tropical Latin America:** Brazil, Paraguay
**North Africa and Middle East**	**North Africa and Middle East:** Afghanistan, Algeria, Bahrain, Egypt, Iran, Iraq, Jordan, Kuwait, Lebanon, Libyan Arab Jamahiriya, Morocco, Occupied Palestinian Territory, Oman, Qatar, Saudi Arabia, Sudan, Syrian Arab Republic, Tunisia, Turkey, United Arab Emirates, Yemen
**South Asia**	**South Asia:** Bangladesh, Bhutan, India, Nepal, Pakistan
**Southeast Asia, East Asia and Oceania**	**East Asia:** China, Dem. People´s Republic of Korea, Taiwan<br>**Southeast Asia:** Cambodia, Indonesia, Lao People´s Democratic Republic, Malaysia, Maldives, Mauritius, Myanmar, Philippines, Seychelles, Sri Lanka, Thailand, Timor-Leste, Vietnam<br>**Oceania:** Fiji, Kiribati, Marshall Islands, Micronesia, Papua New Guinea, Samoa, Solomon Islands, Tonga, Vanuatu
**Sub-Saharan Africa**	**Central Sub-Saharan Africa:** Angola, Central African Republic, Congo, Democratic Republic of the Congo, Equatorial Guinea, Gabon<br>**Eastern Sub-Saharan Africa:** Burundi, Comoros, Djibouti, Eritrea, Ethiopia, Kenya, Madagascar, Malawi, Mozambique, Rwanda, Somalia, Uganda, United Republic of Tanzania, Zambia<br>**Southern Sub-Saharan Africa:** Botswana, Lesotho, Namibia, South Africa, Swaziland, Zimbabwe<br>**Western Sub-Saharan Africa:** Benin, Burkina Faso, Côte d´Ivoire, Cameroon, Cape Verde, Chad, Gambia, Ghana, Guinea, Guinea-Bissau, Liberia, Mali, Mauritania, Niger, Nigeria, São Tomé and Príncipe, Senegal, Sierra Leone, Togo

Supplementary Figure 1: Eye health research output per capita 2000-2019.

The size of the bubble reflects the total output in the region; regions within each Super-region are the same colour; prevalence of blindness data from Vision Loss Expert Group[11].

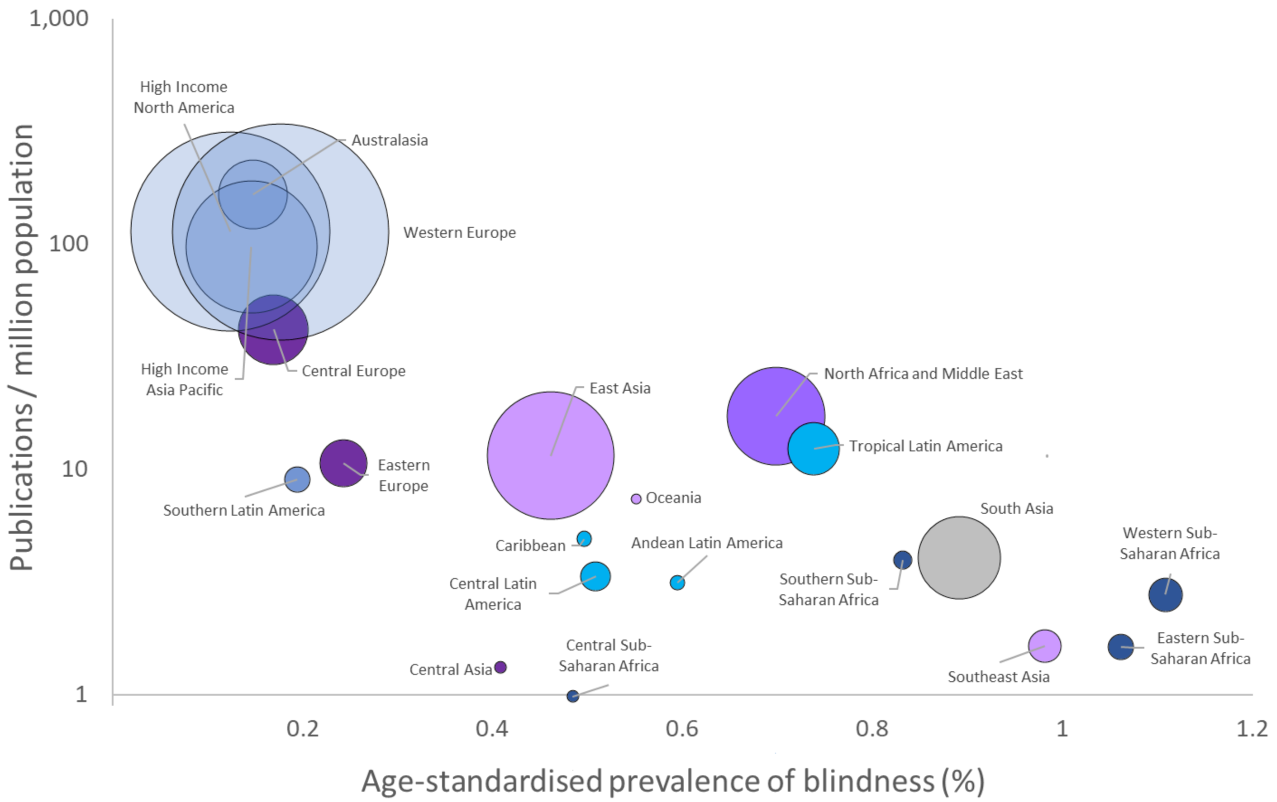

Supplementary Table 2: Eye health research output per capita 2000-2019 (shown in Supplementary Figure 1)

Region	2015 Population (millions)*	Age-Adjusted blindness prevalence 2020**	Number of eye health publications	Eye health publications /million population
Andean Latin America	58.2	0.594549	183	3.1
Australasia	28.5	0.147651	4726	165.8
Caribbean	43.4	0.496365	213	4.9
Central Asia	87.0	0.40837	115	1.3
Central Europe	115.7	0.169224	4826	41.7
Central Latin America	251.7	0.508527	844	3.4
Central Sub-Saharan Africa	111.8	0.484364	110	1.0
East Asia	1431.9	0.461054	16450	11.5
Eastern Europe	208.0	0.242811	2208	10.6
Eastern Sub-Saharan Africa	376.4	1.061295	611	1.6
High Income Asia Pacific	182.9	0.146143	17692	96.7
High Income North America	357.7	0.124008	40624	113.6
North Africa and Middle East	562.1	0.698207	9663	17.2
Oceania	10.0	0.551272	74	7.4
South Asia	1690.3	0.891473	6863	4.1
Southeast Asia	649.9	0.981111	1069	1.6
Southern Latin America	64.8	0.194284	585	9.0
Southern Sub-Saharan Africa	78.2	0.83175	310	4.0
Tropical Latin America	214.5	0.737864	2648	12.3
Western Europe	423.5	0.176577	47790	112.8
Western Sub-Saharan Africa	393.4	1.108342	1093	2.8
**Global**	**7340.0**		158697	21.6

Data provided by the Vision Loss Expert Group: Bourne R, Steinmetz JD, Flaxman S, et al. Trends in prevalence of blindness and distance and near vision impairment over 30 years: an analysis for the Global Burden of Disease Study. *The Lancet Global Health*. 2021;9(2):e130-e143.

Supplementary Table 3: Annual output and average annual change of primary research on eye health by GBD region, 2000-2019 (shown in Figure 3)

Super-region	Region	Year																				Total	Average annual increase
		2000	2001	2002	2003	2004	2005	2006	2007	2008	2009	2010	2011	2012	2013	2014	2015	2016	2017	2018	2019		
High-income	Western Europe	1827	1964	1812	1941	2028	2217	2251	2292	2268	2295	2463	2508	2488	2744	2721	2761	2740	2795	2797	2878	47790	2.5%
	High-income North America	1449	1440	1490	1578	1623	1779	1851	1899	1975	1982	1910	2243	2241	2356	2394	2429	2555	2347	2584	2499	40624	3.2%
	High-income Asia Pacific	599	553	510	530	652	645	697	720	749	831	821	965	1116	1159	1160	1128	1207	1319	1144	1187	17692	5.1%
	Australasia	144	147	157	160	166	188	260	239	239	233	244	297	298	287	274	263	278	285	294	273	4726	3.6%
	Southern Latin America	22	24	22	15	25	20	28	24	20	25	33	17	44	45	27	31	29	36	47	51	585	4.5%
Southeast Asia, East Asia & Oceania	East Asia	246	275	339	334	359	457	478	539	649	702	753	917	1014	1021	1116	1284	1301	1463	1531	1672	16450	10.3%
	Southeast Asia	14	17	28	43	34	48	45	44	66	49	65	69	90	54	59	59	65	71	78	71	1069	5.5%
	Oceania	3	1	1	1	—	2	2	2	5	1	6	8	3	2	6	5	6	9	4	7	74	9.5%
North Africa and Middle East	North Africa and Middle East	197	205	247	268	290	396	395	496	418	488	491	484	532	611	596	595	757	677	710	810	9663	6.6%
Central Europe, Eastern Europe & Central Asia	Central Europe	177	175	208	220	300	259	248	286	248	191	238	247	272	272	280	248	265	213	216	263	4826	0.9%
	Eastern Europe	98	112	121	127	96	100	92	90	111	96	96	124	110	115	103	99	115	108	148	147	2208	1.1%
	Central Asia	3	1	3	6	4	3	7	5	5	5	7	7	2	5	6	11	9	8	15	3	115	6.0%
South Asia	South Asia	149	152	178	243	211	208	257	278	298	292	340	357	425	416	433	451	487	508	567	613	6863	7.2%
Latin America & Caribbean	Tropical Latin America	32	37	32	48	81	149	175	194	185	187	163	157	163	172	140	155	122	146	148	162	2648	4.4%
	Central Latin America	18	18	27	22	24	43	33	48	42	47	45	44	39	50	53	54	58	50	55	74	844	5.6%
	Caribbean	8	20	8	8	12	8	3	12	10	9	12	14	11	14	12	6	16	11	13	6	213	0.4%
	Andean Latin America	2	1	3	6	3	8	5	9	9	8	9	11	10	12	8	17	16	16	13	17	183	9.3%
Sub-Saharan Africa	Western	29	35	34	38	34	47	35	58	53	70	65	55	62	59	77	53	75	66	74	74	1093	4.5%
	Eastern	14	22	11	17	19	16	23	23	27	26	43	46	34	42	42	38	41	41	44	42	611	6.2%
	Southern	17	16	15	12	12	11	14	14	13	10	14	12	21	16	19	19	24	21	11	19	310	2.0%
	Central	9	5	7	4	7	4	5	7	1	8	4	1	7	4	8	6	5	4	8	6	110	-0.3%
	**Total**	**5057**	**5220**	**5253**	**5621**	**5980**	**6608**	**6904**	**7279**	**7391**	**7555**	**7822**	**8583**	**8982**	**9456**	**9534**	**9712**	**10171**	**10194**	**10501**	**10874**	**158697**	**4.2%**

Supplementary Table 4: Main condition of primary research on eye health by year, 2000-2019 (shown in Supplementary Figure 2)

	Condition					
**Year**	**Glaucoma**	**Cataract**	**Refractive Error**	**Diabetic retinopathy**	**ARM**	**Other+**
2000	573	624	439	324	183	2914
2001	552	597	472	329	192	3078
2002	561	626	520	316	234	2996
2003	622	620	550	346	264	3219
2004	661	629	599	401	309	3381
2005	696	670	649	420	340	3833
2006	813	712	652	425	410	3892
2007	767	775	684	461	490	4102
2008	824	727	681	512	524	4123
2009	799	730	712	516	525	4273
2010	849	714	675	532	508	4544
2011	1009	768	748	570	616	4872
2012	969	784	788	618	587	5236
2013	1019	826	901	643	636	5431
2014	1079	790	790	652	718	5505
2015	1071	843	820	689	747	5542
2016	1153	869	782	785	754	5828
2017	1126	815	812	838	689	5914
2018	1123	826	859	793	701	6199
2019	1143	771	825	859	657	6619
**Total n**	**17409**	**14716**	**13958**	**11029**	**10084**	**91501**
**%**	**11.0**	**9.3**	**8.8**	**6.9**	**6.4**	**57.7**

Supplementary Figure 2: Main condition of primary research on eye health by year, 2000-2019

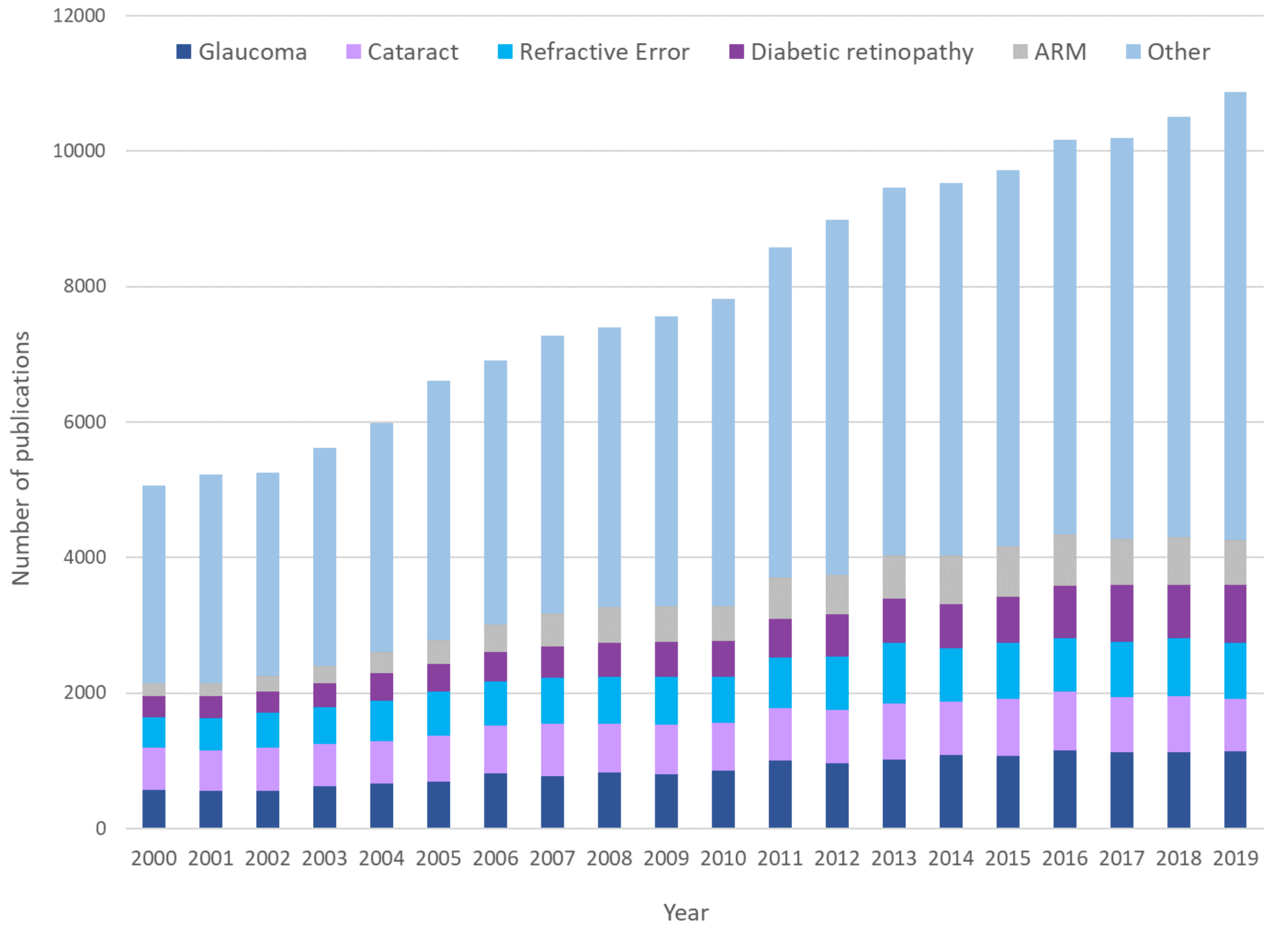

Supplementary Table 5: Main condition of primary research on eye health by GBD region, 2000-2019 (shown in Figure 3)

GBD Super-Region	Condition								Total
	Cataract	Refractive Error	Glaucoma	ARM	Diabetic retinopathy	Corneal	Trachoma	Other	
Central Europe, Eastern Europe & Central Asia	861	604	959	370	537	375	23	3420	7149
High-income	9147	9405	12473	8349	7355	6311	250	58127	111417
Latin America & Caribbean	358	266	355	126	283	290	31	2179	3888
North Africa and Middle East	851	846	843	313	693	764	65	5288	9663
South Asia	1227	528	843	176	562	595	45	2887	6863
Southeast Asia, East Asia & Oceania	2032	2141	1720	731	1519	983	50	8417	17593
Sub-Saharan Africa	240	168	216	19	80	61	381	959	2124

Supplementary Table 6a: Proportion of all authors assigned a gender who were women, by region of research and year of publication (shown in Figure 4)

	Central Europe, Eastern Europe, and Central Asia			High-income			Latin America and Caribbean			North Africa and Middle East			South Asia			Southeast Asia, East Asia, and Oceania			Sub-Saharan Africa			Global		
Year*	Total	Female		Total	Female		Total	Female		Total	Female		Total	Female		Total	Female		Total	Female		Total**	Female	
	n	n	%	n	n	%	n	n	%	n	n	%	n	n	%	n	n	%	n	n	%	n	n	%
2002	877	322	37	16824	4575	27	322	89	28	815	232	28	737	176	24	1645	507	31	195	34	17	21415	5935	28
2003	1016	348	34	18297	5128	28	329	109	33	927	241	26	989	232	23	1638	469	29	234	38	16	23430	6565	28
2004	1353	651	48	19643	5490	28	542	157	29	1027	282	27	835	189	23	1737	564	32	263	68	26	25400	7401	29
2005	1274	504	40	21857	6438	29	935	336	36	1532	432	28	886	207	23	2279	676	30	260	62	24	29023	8655	30
2006	1225	533	44	23468	6790	29	956	349	37	1470	471	32	1014	234	23	2539	773	30	286	76	27	30958	9226	30
2007	1364	572	42	25040	7434	30	1239	455	37	1915	560	29	1190	293	25	2974	892	30	329	78	24	34051	10284	30
2008	1207	451	37	24935	7558	30	1268	436	34	1728	570	33	1204	295	25	3631	1071	29	324	87	27	34297	10468	31
2009	1078	423	39	26213	8081	31	1305	485	37	2164	668	31	1192	308	26	3925	1233	31	446	125	28	36323	11323	31
2010	1322	507	38	28368	8935	31	1141	415	36	2297	644	28	1522	401	26	4481	1459	33	524	129	25	39655	12490	31
2011	1523	657	43	32819	10469	32	1150	452	39	2414	763	32	1703	459	27	5600	1792	32	534	145	27	45743	14737	32
2012	1628	688	42	34984	11492	33	1218	456	37	2620	771	29	1958	584	30	6000	1961	33	521	123	24	48929	16075	33
2013	1714	711	41	38142	12826	34	1382	551	40	2994	878	29	1961	492	25	6365	2129	33	607	198	33	53165	17785	33
2014	1769	695	39	38929	13374	34	1259	492	39	2960	906	31	2133	642	30	7067	2439	35	755	240	32	54872	18788	34
2015	1702	724	43	40392	14089	35	1422	593	42	3226	1040	32	2433	752	31	8074	2753	34	678	202	30	57927	20153	35
2016	1855	853	46	43128	15359	36	1234	545	44	3947	1320	33	2415	777	32	8218	2786	34	952	290	30	61749	21930	36
2017	1742	808	46	43958	15581	35	1426	638	45	3556	1302	37	2676	898	34	9380	3210	34	728	208	29	63466	22645	36
2018	1896	828	44	44544	16151	36	1451	642	44	3768	1414	38	2996	1013	34	9915	3507	35	959	277	29	65529	23832	36
2019	1886	783	42	41790	15258	37	1553	678	44	3967	1468	37	3090	1096	35	10363	3775	36	882	286	32	63531	23344	37
Total	26431	11058	42	563331	185028	33	20132	7878	39	43327	13962	32	30934	9048	29	95831	31996	33	9477	2666	28	789463	261636	33

* MEDLINE began to record full names of all authors from 2002, so the analysis on gender excluded articles published in 2000 and 2001.

**Gender assigned by gender-api to 89% of all authorships; those unable to be assigned excluded from this table.

Supplementary Table 6b: Proportion of all first authors assigned a gender who were women, of research and year of publication (shown in Figure 4)

	Central Europe, Eastern Europe, and Central Asia			High-income			Latin America and Caribbean			North Africa and Middle East			South Asia			Southeast Asia, East Asia, and Oceania			Sub-Saharan Africa			Global		
Year*	Total	Female		Total	Female		Total	Female		Total	Female		Total	Female		Total	Female		Total	Female		Total**	Female	
	n	n	%	n	n	%	n	n	%	n	n	%	n	n	%	n	n	%	n	n	%	n	n	%
2002	246	94	38	3345	1032	31	67	21	31	180	50	28	154	41	27	332	106	32	57	7	12	4381	1351	31
2003	292	103	35	3568	1090	31	70	29	41	209	53	25	208	44	21	344	100	29	58	7	12	4749	1426	30
2004	338	158	47	3733	1148	31	106	38	36	221	57	26	179	39	22	373	118	32	54	9	17	5004	1567	31
2005	305	122	40	4060	1323	33	200	80	40	323	95	29	169	44	26	466	135	29	60	14	23	5583	1813	32
2006	282	115	41	4308	1383	32	201	76	38	299	96	32	220	54	25	478	163	34	63	15	24	5851	1902	33
2007	304	138	45	4487	1496	33	251	97	39	403	114	28	226	54	24	546	166	30	81	20	25	6298	2085	33
2008	287	111	39	4412	1511	34	231	81	35	343	112	33	238	59	25	654	203	31	71	15	21	6236	2092	34
2009	243	100	41	4613	1631	35	235	93	40	406	128	32	234	59	25	682	240	35	86	23	27	6499	2274	35
2010	291	130	45	4782	1685	35	214	79	37	436	107	25	287	85	30	777	293	38	103	27	26	6890	2406	35
2011	328	141	43	5453	1971	36	207	87	42	447	139	31	313	74	24	938	331	35	101	20	20	7787	2763	35
2012	331	165	50	5631	2086	37	214	74	35	493	127	26	364	117	32	1030	367	36	108	23	21	8171	2959	36
2013	348	159	46	6052	2277	38	229	97	42	554	173	31	363	96	26	1003	346	34	105	34	32	8654	3182	37
2014	339	132	39	6052	2320	38	197	85	43	538	165	31	403	132	33	1085	403	37	132	44	33	8746	3281	38
2015	308	140	45	6077	2372	39	220	94	43	541	174	32	422	136	32	1239	441	36	101	34	34	8908	3391	38
2016	342	178	52	6276	2548	41	197	96	49	707	240	34	447	170	38	1231	447	36	129	48	37	9329	3727	40
2017	305	161	53	6296	2501	40	210	109	52	634	252	40	470	160	34	1352	509	38	121	39	32	9388	3731	40
2018	338	171	51	6459	2634	41	218	96	44	675	253	37	533	212	40	1435	578	40	120	36	30	9778	3980	41
2019	348	162	47	5964	2480	42	220	105	48	722	265	37	537	200	37	1500	547	36	124	42	34	9415	3801	40
Total	5575	2480	44	91568	33488	37	3487	1437	41	8131	2600	32	5767	1776	31	15465	5493	36	1674	457	27	131667	47731	36

* MEDLINE began to record full names of all authors from 2002, so the analysis on gender excluded articles published in 2000 and 2001.

**Gender assigned by gender-api to 89% of all authorships; those unable to be assigned excluded from this table.

Supplementary Table 6c: Proportion of all last authors assigned a gender who were women, of research and year of publication (shown in Figure 4)

	Central Europe, Eastern Europe, and Central Asia			High-income			Latin America and Caribbean			North Africa and Middle East			South Asia			Southeast Asia, East Asia, and Oceania			Sub-Saharan Africa			Global		
Year*	Total	Female		Total	Female		Total	Female		Total	Female		Total	Female		Total	Female		Total	Female		Total**	Female	
	n	n	%	n	n	%	n	n	%	n	n	%	n	n	%	n	n	%	n	n	%	n	n	%
2002	251	82	33	3323	596	18	63	12	19	179	49	27	150	26	17	325	88	27	47	9	19	4338	862	20
2003	288	78	27	3475	651	19	69	20	29	206	52	25	202	40	20	335	96	29	47	7	15	4622	944	20
2004	338	152	45	3673	679	18	99	24	24	221	62	28	169	33	20	358	126	35	51	6	12	4909	1082	22
2005	308	101	33	4009	813	20	188	46	24	312	67	21	176	32	18	473	145	31	63	14	22	5529	1218	22
2006	283	106	37	4291	819	19	199	58	29	303	74	24	210	39	19	477	139	29	60	13	22	5823	1248	21
2007	303	121	40	4450	906	20	243	67	28	391	85	22	237	45	19	542	158	29	73	12	16	6239	1394	22
2008	286	88	31	4378	935	21	225	56	25	332	75	23	244	46	19	649	146	22	68	16	24	6182	1362	22
2009	240	76	32	4575	969	21	222	74	33	404	82	20	245	55	22	675	190	28	83	23	28	6444	1469	23
2010	287	90	31	4744	1015	21	215	67	31	442	88	20	283	49	17	755	208	28	101	19	19	6827	1536	22
2011	338	132	39	5335	1235	23	205	64	31	438	106	24	299	70	23	909	236	26	91	21	23	7615	1864	24
2012	333	102	31	5553	1248	22	213	80	38	480	100	21	366	64	17	982	262	27	104	23	22	8031	1879	23
2013	341	117	34	5901	1403	24	227	69	30	553	120	22	363	69	19	957	288	30	108	39	36	8450	2105	25
2014	337	94	28	5936	1404	24	199	51	26	534	129	24	407	91	22	1036	310	30	129	46	36	8578	2125	25
2015	308	103	33	5918	1491	25	216	85	39	543	152	28	403	116	29	1197	338	28	98	18	18	8683	2303	27
2016	339	115	34	6138	1521	25	195	69	35	702	195	28	438	102	23	1207	353	29	122	33	27	9141	2388	26
2017	304	100	33	6144	1608	26	205	74	36	630	184	29	469	118	25	1338	377	28	115	32	28	9205	2493	27
2018	336	102	30	6337	1711	27	213	89	42	670	230	34	512	153	30	1406	368	26	108	27	25	9582	2680	28
2019	340	105	31	6094	1693	28	233	88	38	701	242	35	542	157	29	1497	447	30	121	37	31	9528	2769	29
Total	5560	1864	34	90274	20697	23	3429	1093	32	8041	2092	26	5715	1305	23	15118	4275	28	1589	395	25	129726	31721	24

* MEDLINE began to record full names of all authors from 2002, so the analysis on gender excluded articles published in 2000 and 2001.

**Gender assigned by gender-api to 89% of all authorships; those unable to be assigned excluded from this table.

Supplementary Table 6d: Average annual change in proportion of all authors assigned a gender who were women, 2002-2019 (shown in Figure 4)

Super-region	Average annual change in proportion of female authors		
	All	First	Last
Central Europe, Eastern Europe & Central Asia	0.25%	0.64%	-0.27%
High-income	0.58%	0.68%	0.56%
Latin America & Caribbean	0.80%	0.78%	0.93%
North Africa and Middle East	0.56%	0.64%	0.60%
South Asia	0.79%	1.01%	0.66%
Southeast Asia, East Asia & Oceania	0.40%	0.48%	-0.01%
Sub-Saharan Africa	0.61%	1.21%	0.79%
**Global**	**0.54%**	**0.64%**	**0.49%**

Supplementary Table 7: Proportion of research teams who are women based on the gender of last author across GBD super-regions, primary eye health research 2002-2019 (n=128,167 publications). (shown in Figure 5)

Super-region	Last author male					Last author female				
	Number of outputs	% of region	Median	Inter-quartile range	Range	Number of outputs	% of region	Median	Inter-quartile range	Range
Central Europe, Eastern Europe & Central Asia	3696	66	13	0-43	0-90	1864	34	75	50-100	6-100
High-income	69577	77	22	0-40	0-90	20697	23	50	38-67	6-100
Latin America & Caribbean	2336	68	25	0-50	0-89	1093	32	60	43-75	10-100
North Africa and Middle East	5949	74	20	0-38	0-86	2092	26	50	40-75	10-100
South Asia	4410	77	20	0-33	0-86	1305	23	50	33-67	10-100
Southeast Asia, East Asia & Oceania	10843	72	25	0-40	0-89	4275	28	50	33-63	10-100
Sub-Saharan Africa	1194	75	0	0-29	0-83	395	25	50	37-67	11-100
**Total**	98005	76	20	0-40	0-90	31721	24	50	38-71	6-100

Supplementary Table 8: Geographic distribution of research with authors with unknown gender and

GBD Region	Total Authors	Unknown gender		Proportion female among predicted gender	Proportion female (%) if unknown gender were:		
		n	%	(presented results, %)	All Male	All Female	50% Female
Central Sub-Saharan Africa	569	143	25.1	28.6	21.4	46.6	34.0
Southern Sub-Saharan Africa	1182	238	20.1	34.9	27.8	48.0	37.9
Andean Latin America	1216	240	19.7	40.3	32.3	52.1	42.2
Western Sub-Saharan Africa	5806	1117	19.2	24.7	19.9	39.2	29.6
Central Europe	22121	4146	18.7	53.8	43.7	62.5	53.1
Central Asia	503	93	18.5	27.8	22.7	41.2	31.9
Southeast Asia	5285	930	17.6	42.0	34.6	52.2	43.4
Western Europe	275325	48389	17.6	36.1	29.8	47.4	38.6
Caribbean	1126	185	16.4	40.6	33.9	50.4	42.1
North Africa and Middle East	50030	6703	13.4	32.2	27.9	41.3	34.6
Central Latin America	4602	606	13.2	35.4	30.7	43.9	37.3
Southern Latin America	3587	450	12.5	43.8	38.3	50.8	44.5
South Asia	35099	4165	11.9	29.2	25.8	37.6	31.7
Eastern Sub-Saharan Africa	3860	442	11.5	30.9	27.4	38.8	33.1
East Asia	100732	9671	9.6	33.0	29.8	39.4	34.6
Australasia	24577	1786	7.3	35.5	32.9	40.2	36.5
High-income Asia Pacific	104015	7099	6.8	20.9	19.5	26.3	22.9
Oceania	443	28	6.3	33.5	31.4	37.7	34.5
High-income North America	225202	11651	5.2	34.3	32.5	37.7	35.1
Eastern Europe	8449	403	4.8	15.8	15.1	19.8	17.4
Tropical Latin America	14929	710	4.8	40.0	38.1	42.9	40.5
**Total**	888658	99195	11.2	**33.1**	**29.4**	**40.6**	**35.0**
